# *In Vitro* Evaluation of the Effect of NaOCl Deproteinization of MIH-Affected Hard Dental Tissue on the Marginal Integrity of a Glass Hybrid Material

**DOI:** 10.3390/ma19020229

**Published:** 2026-01-07

**Authors:** Agata Ćwiklińska, Joanna Szczepańska, Joanna Nowak, Sylwia Majewska-Beśka, Agnieszka Bruzda-Zwiech

**Affiliations:** 1 Department of Paediatric Dentistry, Medical University of Lodz, 92-213 Lodz, Poland; agata.cwiklinska@umed.lodz.pl (A.Ć.); joanna.szczepanska@umed.lodz.pl (J.S.); sylwia.majewska-beska@umed.lodz.pl (S.M.-B.); 2 University Laboratory of Materials Research, Medical University of Lodz, 92-213 Lodz, Poland; joanna.nowak.1@umed.lodz.pl

**Keywords:** molar hypomineralization, developmental enamel defects, molar–incisor hypomineralization (MIH) deproteinization, sodium hypochlorite, glass ionomer cements, marginal adaptation

## Abstract

The enamel of teeth affected by Molar–Incisor Hypomineralization (MIH) has been reported to have a higher protein content. Though a glass hybrid is recommended for restoring teeth with MIH in children, there is a lack of in vitro research on the influence of deproteinization on its marginal integrity. Therefore, this study aimed to evaluate whether enamel pretreatment with 5.25% NaOCl reduces the size of the marginal crevice of such restorations. Out of eight extracted teeth with severe MIH, restored using a glass hybrid (Equia Forte HT/GC), half underwent deproteinization. A stereoscopic and a scanning electron microscope (SEM) were used for sections analysis. The median value of the marginal crevice measured using stereoscopic microscopy (n = 17) was significantly lower for the deproteinized (6.78 μm) than for the standard-prepared specimens (12.61 μm), *p* = 0.008. On SEM images, the median marginal crevice (n = 10) was 69.40 μm versus 156.77 μm for the deproteinized and standard groups, respectively. The differences, however, were not statistically significant. This study only partially confirmed the hypothesis that pretreatment with NaOCl reduces marginal crevices between the Equia Forte HT material and hypomineralized hard tissues. Further studies on the effect of deproteinization on the marginal adaptation of glass hybrid materials are needed.

## 1. Introduction

Molar–Incisor Hypomineralization (MIH) is a qualitative developmental defect of enamel, with a multifactorial and not fully elucidated etiology [1]. It affects one or more permanent molars, and frequently incisors are involved [2,3,4]. The newest systematic review and meta-analysis confirmed that MIH has become a global problem, with the average prevalence in children worldwide estimated at 15.5% (14.4–16.5%) and ranging from 0.6 to 46.6%. MIH prevalence varies across continents, with the highest reported in North America (23.9%), followed by South America (17.1%), while in Europe, it was calculated at 15.4% [5]. The underlying cause of the varying MIH prevalence rates across continents is a complex issue that can be affected by inconsistent diagnostic criteria, varied sample sizes, different study quality, heterogeneity for the age of the examined patients, and diverse populations (e.g., ethnic population affecting generalizability of the results), as well as due to a mix of local environmental (regional deviations—urban or rural communities, industrialization, disease burdens, pollutants exposures, etc.) or genetic factors [5,6,7]. Teeth affected by MIH show clearly demarcated opacities from creamy/white through to yellow or brown color. MIH defects may have different grades of severity (from mild to severe). The clinical picture of severe MIH includes post-eruptive enamel breakdown (PEB) and rapidly progressing caries, causing atypical restorations, which are accompanied by spontaneous and persistent hypersensitivity affecting functions such as brushing and mastication [1]. The increased enamel porosity, its lowered hardness, and modules of elasticity—that cause decreased mechanical resistance to occlusal loads—can be explained by alteration in the enamel build of MIH-affected teeth [8].

The enamel of teeth with hypomineralization is characterized by an irregular and disorganized structure without distinct prisms borders and reduced hydroxyapatite content, which increases its porosity. There are also large interprismatic spaces occupied by a protein-rich matrix [8,9,10]. The protein content of MIH-affected enamel has been reported to be significantly higher (between 3 and 21 times) than that of healthy enamel, which contributes to the inhibition of hydroxyapatite crystal growth, resulting in impaired mineralization [8,11]. The chemical microanalysis of the mineral composition of hypomineralized enamel showed a different distribution of minerals, with the percentages of calcium and phosphorus lower than in healthy enamel. The carbon and carbonate contents were higher than physiologically (up to 10% compared with 3% in normal enamel), which may indicate persistence of remains of organic matter [8,12]. The mean mineral density was reported to be 19–20% lower compared to that of normal enamel. High protein and low mineral content have been identified to be risk factors for PEB, especially in yellow/brown enamel opacities [8].

Modern restorative dentistry relies on the adhesion of materials to the dental hard tissues. Quantitative changes in the enamel of teeth affected by MIH may prevent optimal and predictable adhesion to resin composite [13]. Acid-etched MIH enamel has more cracks, deep pores, and shows abnormal etching patterns [8,12], making it impossible to obtain typical etching patterns with the characteristic appearance of a honeycomb (type 1) or a cobblestone effect (type 2) [12]. The mean microshear bond strengths (SBSs) of resin bonded to hypomineralized enamel were found to be significantly lower than those bonded to sound enamel [14]. To improve the adhesion of resin composite restorations in molars with MIH, some authors advise the removal of all defective enamel until sound enamel surfaces are reached. However, this invasive approach leads to major tooth surface loss [3,13]. Another suggested approach is the removal of defective enamel only until resistance to the bur or to the probe is felt [15,16]. Sönmez and Saat [17] showed that resin composite restorations were more likely to fail in MIH-affected teeth where the cavity surrounding tissue was hypomineralized. Data from the literature showed that a deproteinization procedure with 5% NaOCl may improve adhesion to developmentally hypomineralized tissue and enhanced the retention rate of resin composites [17,18]. However, some in vitro data showed that an additional pretreatment of altered enamel with NaOCl did not enhance enamel bonding [13,19].

The management of MIH in children is especially challenging due to the difficulty in achieving local analgesia in MIH-affected molar teeth with hypersensitivity, which is probably caused by subclinical pulpitis and changes in pulpal blood flow [20]. Behavioral problems and children’s shorter attention span can also pose a difficulty [21]. These factors can limit the possibility of introducing proper adhesive application protocol, tissue preparation, and restoration with resin composites. Additionally, due to the extensive sensitivity of teeth affected with severe MIH, teeth brushing is a painful experience, which makes oral hygiene in these children defective. This encourages the development of primary and secondary caries [1,22]. The above factors, as well as the need for minimally invasive procedures for children, seem to prompt the use of new-generation glass ionomers, such as the glass hybrid restorative system Equia Forte HT (GC, Tokyo, Japan) [23].

Glass ionomer cements (GICs) have been widely used in pediatric dentistry thanks to their biocompatibility and chemical adhesion to enamel and dentin [24]. The prevention of secondary caries by fluoride gradually released from GICs is an additional advantage [25]. GICs are recommended for MIH-affected molars in children, to maintain a less invasive approach preserving hypomineralized molars’ integrity, until the child is mature enough for more complex procedures [7,26]. Equia Forte, a bulk-fill reinforced highly viscous glass ionomer cement, was designed for load–stress areas in posterior regions. This glass hybrid powder is made of fluoroaluminosilicate glass reinforced with ultrafine, highly reactive silicate type glass particles. The inclusion of high-molecular-weight poly(acrylic acid) further improved its the mechanical properties. [23,27,28]. The particle size distribution in Equia Forte HT has been further optimized compared to its predecessor (Equia Forte), resulting in an increase in the flexural and compressive strength [28]. The material can be covered with a resin-based coat (containing a multifunctional monomer and silica fillers) to improve wear resistance [28]. An in vitro study of Singh et al. [29] showed that Equia Forte exhibited good marginal integrity and better performance in limiting microleakage around the restoration margins than Fuji II LC (GC Corporation, Tokyo, Japan) and GC G-aenial anterior resin composite (GC Corporation, Tokyo, Japan). Equia Forte also proved to be successful in class I and class II cavities and in hypomineralized teeth [23,30,31,32]. Sezer et al. showed that over a six-year follow-up period, the overall estimated mean survival time of glass hybrid restorations in severely hypominerlized molar teeth was 59.82 ± 1.50 months [33]. Additionally, the manufacturer claims that the material does not require preconditioning of the cavity for sufficient bonding.

Due to the increased incidence of MIH in children and adolescents, research is needed on the techniques that may improve adhesion of new-generation glass ionomer materials to altered hard dental tissues and reduce the failure of their marginal sealing. In teeth with severe enamel developmental defects, the adhesion to altered enamel may not be the only challenge. In severe MIH, the dentin under the hypomineralized enamel can also present a higher level of particles related to organic matter than in normal dentin [34]. It may not undergo chemical modification by acids to the same extent as healthy tissue. The action of high-concentration hypochlorite is to remove excess organic matter, especially proteins, which adversely affect the adhesion to developmentally altered tissues [8,17,19]. However, in the literature, there are no data on the effect of deproteinization of dental tissue affected by hypomineralization on the marginal integrity of a glass hybrid. Therefore the study was aimed at microscopic evaluation of the structure of altered tissues in MIH-affected first molar teeth and evaluation as to whether a deproteinization protocol with the use of 5.25% NaOCl reduces the size of the marginal crevice of glass hybrid restorations.

## 2. Materials and Methods

The study was part of a larger research project on the treatment modalities of MIH-affected teeth, approved by the Medical University of Lodz Bioethics Committee, Resolution Number RNN 24/22/KE, and the extension to the ethic approval KE/577/22.

Eight extracted human permanent first molars affected by severe MIH were used for the study. The teeth were qualified for extraction due to justified reasons after thorough dental examination and establishing an individual patient treatment plan. Prior to extraction, parents/legal guardians of adolescent patients were informed about the use of the molars for research purposes, and informed consent was obtained.

Inclusion criteria for the in vitro study were as follows:

- Written consent to use extracted tooth for the study.
- Extracted first permanent molars affected with MIH with uncertain prognosis, obtained from children aged 8–10 years, qualified for extraction due to PEB and associated deep caries (not extending to the pulp) causing significant destruction of the clinical crown.
- Additionally, the tooth status after the extraction procedure needed to allow for cavity preparation and restoration with glass ionomer cement (no tooth fractures caused by the extraction).

**Figure 1:**
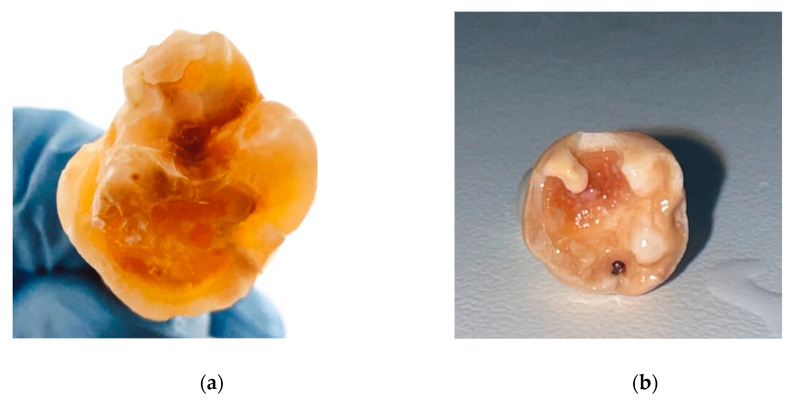
Extracted first permanent molar affected by severe MIH: (**a**) Status after removal of an extensive glass ionomer restoration; (**b**) initial clinical picture of extensive cavity with tooth structure loss and deep caries of uncertain prognosis.

The developmental hypomineralization defects of extracted teeth were classified as severe MIH, characterized by extensive post-eruptive enamel breakdown, destruction of the clinical crown, and deep caries associated with affected hard tissue (Figure 1).

Extracted teeth were cleaned and stored in a 2% sodium azide solution (Merck. Sigma-Aldrich, Poznan, Poland) for no longer than 6 weeks.

**Figure 2:**
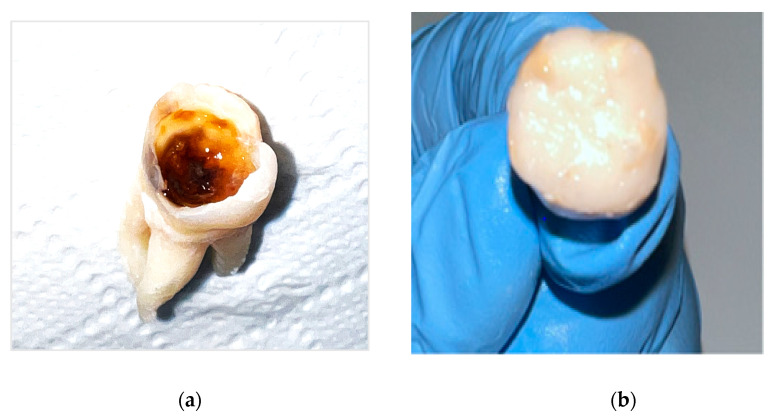
Extracted first molar affected by severe MIH: (**a**) after cavity preparation; (**b**) immediately after restoring with Equia Forte and petroleum jelly coating.

After removal of leaking restorations and caries tissue, cavities were washed with distilled water and dried with a sterile cotton ball. A half of the teeth (n = 4) underwent deproteinization of enamel and dentin surface: 5.25% NaOCl solution was actively applied with a microbrush for 4 min followed by water spray washing for 30 s and drying with a sterile cotton ball (deproteinized group). The other half of the teeth remained non-deproteinized (control group). All teeth in both groups were then filled with the glass hybrid—Equia Forte HT Fil (GC Corporation, Tokyo, Japan, composition; powder: fluoroaluminosilicate glass, poly(acrylic acid), pigment; liquid: water, poly(acrylic acid), carboxylic acid). The glass hybrid was activated in the mixer for 10 s, as indicated by the manufacturer. Immediately thereafter, the capsule was loaded into an applicator, and material was inserted into the cavity in one increment, which was then packed and contoured. The surface of the restorations was protected with petroleum jelly and left for 4 min (Figure 2). Both cavity preparation and restoration were performed by a single operator.

**Figure 3:**
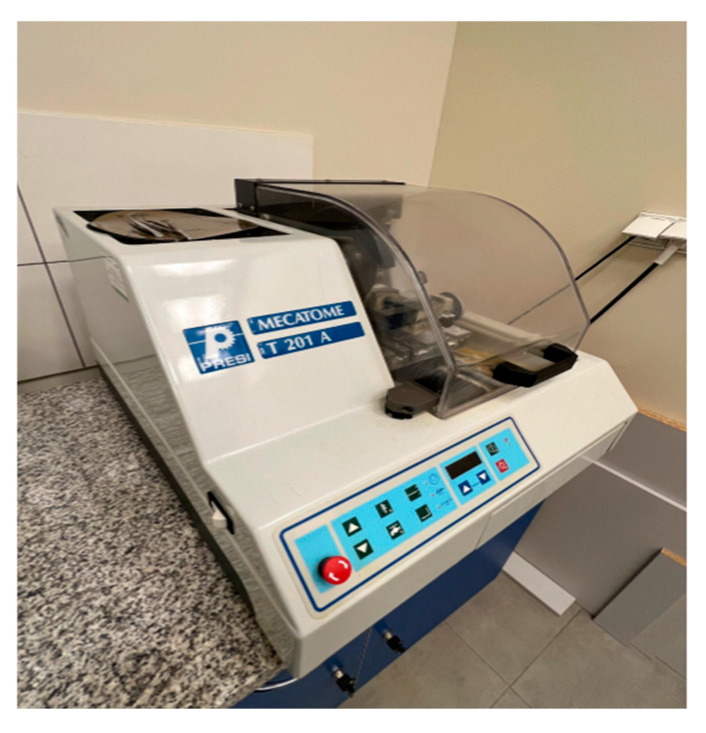
A precision cutter Mecatome T201A (Presi, Eybens, France).

The teeth were cut with a precision cutter Mecatome T201A (Presi, Eybens, France) with water cooling using a diamond-cutting disc. The cut was made along the long axis of the tooth (without cutting off the roots) to reach the vestibular and lingual surfaces, obtaining eight specimens for each group (study and control). The specimens were further processed on a grinder-polishing machine Minitech-233 (Presi, Eybens, France) using P600-graded papers with a silicon carbide (SiC) coating under water cooling (Figure 3).

**Figure 4:**
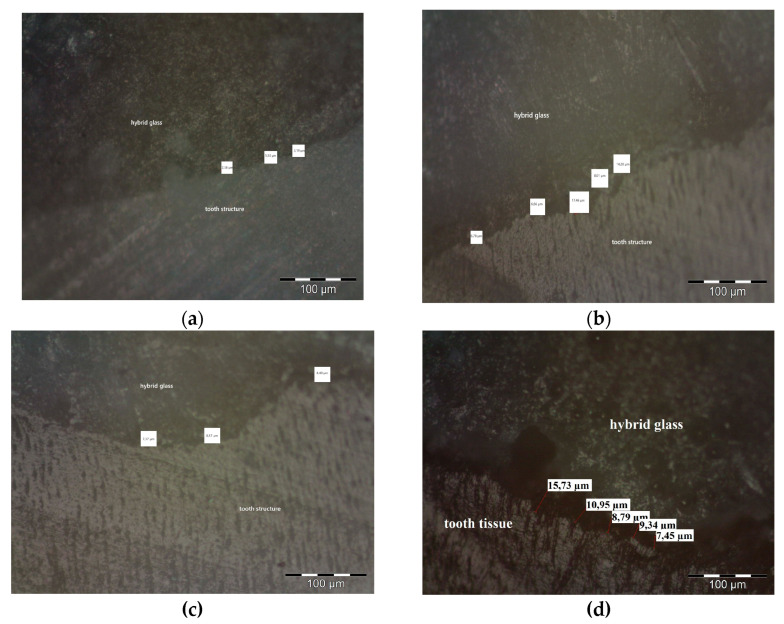
The Equia Forte material and MIH-affected tooth structure interface. Image captured using the BX51 microscope (Olympus, Tokyo, Japan), magnification 100×. (**a**,**b**) Sample after deproteinization; (**c**,**d**) standard prepared sample (without deproteinization).

In order to evaluate the marginal crevice formed at the dental hard tissues and the glass ionomer filling interface, the observations were performed twofold. Each of the 6 (deproteinzed and non-deproteinzed group) specimens were examined using brightfield (BF) observation in an optical microscope (stereoscopic metallographic microscope with Nomarski contrast, BX51, Olympus, Tokyo, Japan) at 100× magnification to evaluate the resulting marginal crevice and the structure of the glass ionomer restoration (Figure 4). Next, a magnification of 200× was used to evaluate the structure of the enamel and dentin, as well as the presence of fractures and cohesion/adhesion cracking. For each group (deproteinized and non-deproteinized), 17 marginal crevice measurements were obtained.

**Figure 5:**
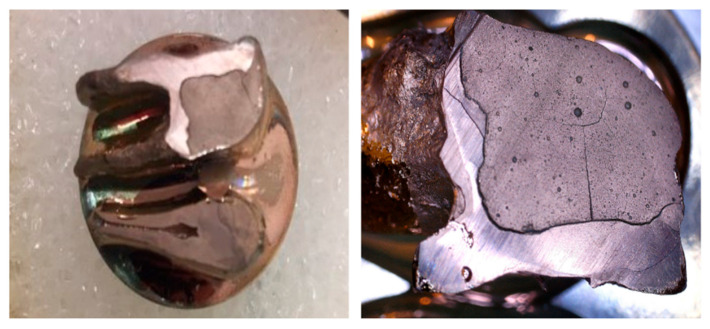
Samples coated with a layer of gold using the sputtering technique prior to SEM examination.

The remaining 2 specimens from each group, after dehydration and gold sputtering (Figure 5), were analyzed using a scanning electron microscope (SEM) with backscattered electron (BSE) detector (JEOL, InTouchScope JSM-IT200, Freising, Germany). An accelerating voltage of 10 kV was used. The images were taken at 18× magnification using the program InTouch Scope v. 1.11 (Smile View^TM^ Map 2.0) to evaluate the structure of enamel and dentin, the presence of fractures, and adhesive and cohesive cracking. For each group of specimens, 10 marginal crevice measurements were taken (5 in each of deproteinized specimens group and 5 in each specimens of prepared in normal manner).

The measurements of marginal crevice were made in selected places where optimal visibility was ensured in the gap between restoration and dentin, trying to capture places with the greatest gap width.

The analyses of the crevice measurements were performed using descriptive statistics—the mean, median, standard deviation, coefficient of variation, range, skewness, and kurtosis were calculated. Statistical analysis was performed with Statistica for Windows software (version 13.3). The Shapiro–Wilk test was used to check for normality of the distribution of the data in particular groups of measurements. To assess the effect of deproteinization on the size of marginal crevice of glass hybrid restorations, the Mann–Whitney U test with the continuity correction was used (the Z statistic with the correction for continuity and for ties). The null hypothesis (Ho) was defined as the following: deproteinization does not affect the size of the marginal crevice (equal medians). The alternative hypothesis (H1) was defined as the following: deproteinization reduces the size of the marginal crevice (lower medians for deproteinized samples). Median values of marginal gap measurements, obtained using a stereoscopic microscope (Olympus, Tokyo, Japan) as well as SEM for deproteinized and non-deproteinized specimens, were compared to determine whether the results support the alternative hypothesis. The *p*-value < 0.05 was considered statistically significant.

## 3. Results

### 3.1. Analysis of Marginal Integrity Between a Glass Hybrid and MIH-Affected Tissue

Of the deproteinized specimens, the largest value of marginal crevice measurement, observed in stereoscopic microscope, was 17.46 μm, and the smallest was 2.58 μm. The median value of the marginal crevice in this group was 6.78 μm. For the standard-prepared specimens, the largest value of the marginal crevice measurement was 50.19 μm, and the minimal was 7.37 μm. The median value of the marginal crevice in the non-deproteinized group was 12.61 μm. Statistically significant differences were observed between those two groups (Z = 2.652, *p* = 0.008). The results are presented in Table 1. (All measurments of marginal crevice between Equia Forte HT glass ionomer material and the tooth surface observed in a stereoscopic microscope are presented in Appendix A, Table A1) 

The largest marginal gap measurement value for the deproteinized specimens was 194.65 μm, and the smallest was 41.07 μm, as examined in SEM.

The median value of marginal crevice measurement in the study group was 69.40 μm. The largest value of marginal crevice measurement for specimens prepared in a standard technique without deproteinization was 591.35 μm, and the smallest value was 41.74 μm. The median value of marginal crevice measurement in that group was 156.77 μm. However, the differences between measurements of marginal crevices between the groups were not statistically significant (Z = 1.550, *p* = 0.121). The results are shown in Table 2. (All measurments of marginal crevice between Equia Forte HT glass ionomer material and the tooth surface observed in SEM are presented in Appendix A, Table A2).

**Figure 6:**
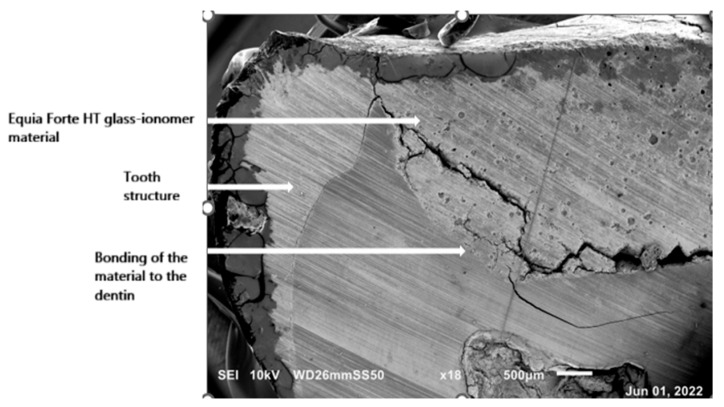
SEM image of an MIH-affected tooth structure prepared in a standard manner. Visible bonding between material and the dentin. Magnification 18×.

**Figure 7:**
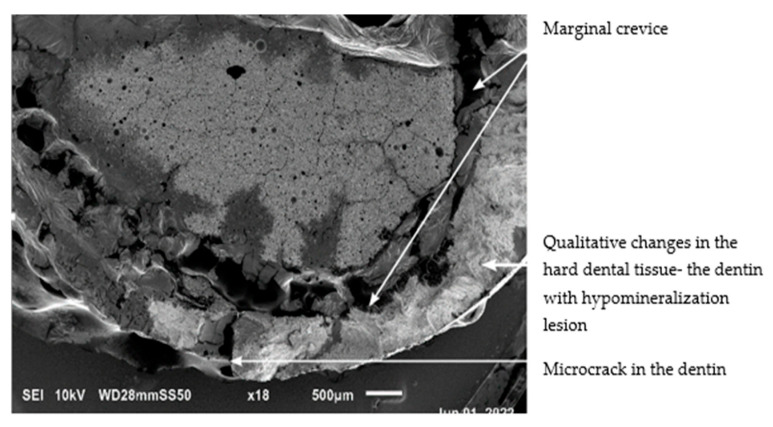
SEM image of an MIH-affected tooth structure prepared in a standard manner. Visible irregular marginal crevice between the tooth and the Equia Forte HT filling. Magnification 18×.

**Figure 8:**
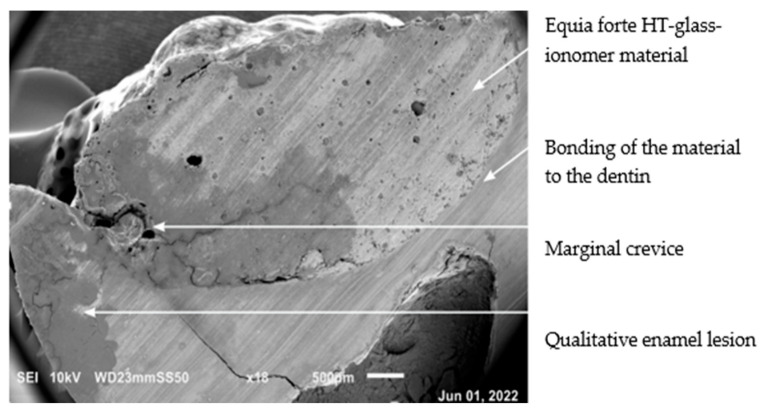
SEM image of a severe MIH-affected tooth structure deproteinized with 5.25% NaOCl. Visible marginal crevice between the glass hybrid material layer and tooth tissue. Qualitative changes in the enamel and dentin. Magnification 18×.

In the studied sections of MIH-affected teeth, larger marginal crevice measurements were obtained from specimens of teeth with the most severe clinical presentation. Prior to laboratory preparation, the MIH-affected teeth were characterized by extensive tissue loss due to multiple corrections of leaking restorations and development of secondary caries, as well as hypomineralization in the junction area with Equia Forte HT glass ionomer material. Figure 6 and Figure 7 presents SEM images of the marginal crevices in samples prepared without deproteinization, and Figure 8 shows the deproteinization protocol.

### 3.2. Analysis of the Enamel and Dentin Structure, the Presence of Fractures, and Cohesion/Adhesion Cracking

**Figure 9:**
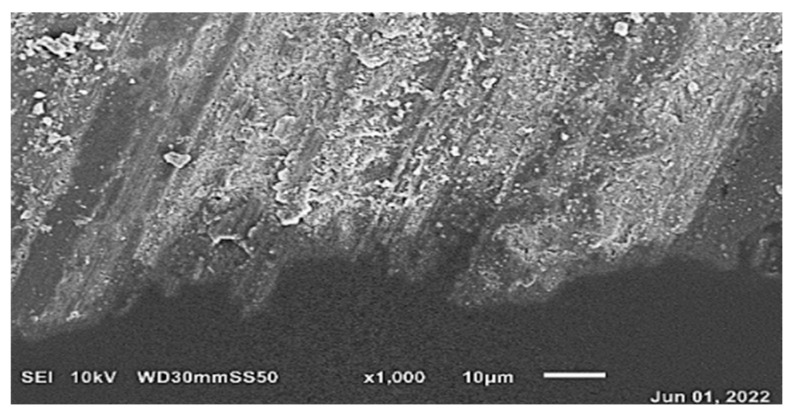
SEM image of an MIH-affected structure of dental hard tissues after deproteinization. Visible partially removed smear layer. Enamel from the area of the dentin–enamel junction. The prisms arranged in irregular pattern. Magnification 1000×.

**Figure 10:**
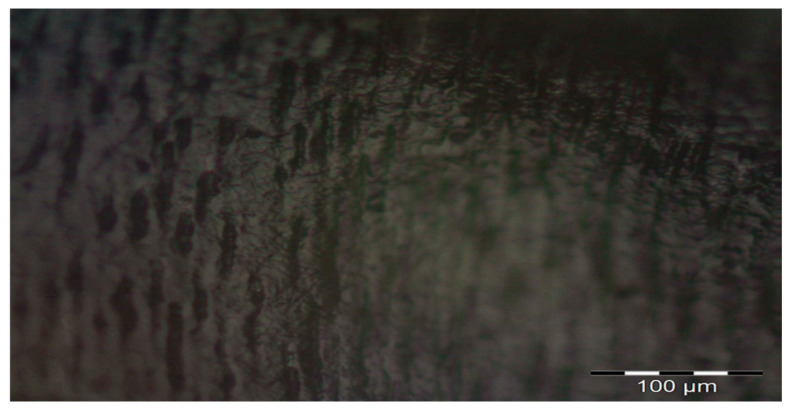
Irregular dentin structure in the area of the enamel–dentin junction of a tooth with hypomineralization. Cross-section. Image from optical microscope. Magnification 100×.

Examination of the hard tissue specimens from teeth with severe MIH using electron microscopy revealed qualitative changes in the structure of the enamel (Figure 6, Figure 8) and dentin (Figure 7). Microscopic analysis of the enamel showed larger interproximal spaces, less regularly arranged apatites, and a deflection of the hard tissue structure present. Locally, the appearance resembled aprismatic enamel typical of deciduous teeth. Examination of the enamel structure using electron microscopy confirmed a disorganized and asymmetrically arranged enamel prism. The deeper the hypomineralization, the more severe clinical manifestation with a change in demarcation color on smooth surfaces was observed. Figure 9 shows the structure of an enamel specimen from the area of the enamel–dentin junction after employing the deproteinization protocol, with a visible partially removed smear layer. The microscopic image of the dentin showed the arrangement of its organic and inorganic structures, which are characteristic for hypomineralization disorders. A disintegration of the regular collagen network was observed, causing an increase in the interglobular spaces. In both the SEM and optical microscope images of the MIH-affected teeth, an irregular pattern of dentinal tubules was found. In the parts closest to the areas with hypomineralization, the dentin lost its tubular appearance and became irregular (Figure 10). In samples deproteinized with a 5.25% solution of NaOCl, the dentin did not show changes in its tubular arrangement (Figure 8). Additionally, in SEM microscopy, fractures and adhesion–cohesion cracks were observed in specimens which had not been prepared with the deproteinization procedure.

## 4. Discussion

To introduce proper prevention and appropriate management of MIH-affected teeth in children, there is a need to understand the nature of these demarcated qualitative developmental defects [8]. Hypomineralization in MIH begins at the dentin–enamel junction (DEJ). In cases of mild MIH, it is limited to the inner third of the enamel. The junction between the hypomineralized internal enamel and the surface enamel is located at the level of one of the striae of Retzius (long-period lines of incremental growth in enamel, which extend from the DEJ to the enamel surface). In contrast, in cases of severe MIH, the entire enamel layer is affected by hypomineralization [35]. Mubarak et al. [36], in their in vitro study, analyzed histological characteristics and the ultrastructure of the altered enamel of first molars affected by hypomineralization. Microscopic images showed opacities varying in extent within the enamel, which were surrounded by fragments of unaltered enamel. The enamel thickness within the opacities was the same as that of a healthy tooth. The opacities followed the lines of Retzius and obscured the characteristic dark and light Hunter–Schreger bands. SEM examination showed irregular and disordered enamel rods with indistinct boundaries, wide interprismatic zones, and loosely packed hydroxyapatite crystals [36]. It has been found microscopically that altered enamel exhibits separation between the interprismatic spaces, creating large (200 nm) gaps where a protein-rich matrix accumulates [35]. It was also shown that the areas of enamel adjacent to the MIH lesion, that appeared clinically normal, had fewer mineralized prism sheaths than unaffected enamel [8].

Also in our study, examination of hard tissue specimens of teeth with severe MIH using an SEM revealed qualitative changes in the structure of the enamel, with larger interproximal spaces and less regularly arranged apatites. Locally, the appearance resembled aprismatic enamel typical for deciduous teeth. Similarly, in the study of Ekambaram et al. [37], SEM analysis showed less distinct prism borders and crystals and greater interprismatic space in the developmentally hypomineralized enamel. These changes, together with reduced mechanical properties due to lowered mineral content and increased protein content—according to the authors—might support the greater number of “cohesive fractures in enamel”. However, the authors observed that the use of deproteinization agents increased fracture resistance and caused a reduction in cohesive enamel fracture after bonding [37]. Similarly, Kramer et al. [13] observed that additional pretreatment of MIH-affected enamel with NaOCl resulted in less pre-test failures, including cohesive and adhesive fractures. Also in our study, fractures and cohesion cracks were observed in specimens which had not been pretreated with NaOCl.

Elhennawy et al. [8], in a systematic review, summarized that observations using scanning electron microscopy or transmission electron microscopy indicate that in enamel affected by MIH, the prism structure is less dense, with a partial loss of prismatic pattern. Furthermore, the crystals are loosely packed, there are less distinct prism borders, more marked interprismatic space, and wider sheath regions. Our observation of the enamel ultra-structure using an SEM also confirms a disorganized pattern of prisms that are asymmetrically arranged. This may result in a loss of mechanical as well as optical properties responsible for aesthetics. The presence of voids between the enamel prisms changes the refractive index of damaged enamel, making it appear more opaque. Additionally, it was proved that the enamel microstructure (prism orientation) has an influence on the adhesion and might influence the enamel bond strength and durability [38].

Data from the literature show that the higher protein content of MIH-affected enamel may play an important role in the pathogenesis of its hypomineralization. Altered enamel shows an over-expression of albumin, calcium-binding proteins, and dentin sialophosphoprotein (DSPP). Additionally, down-regulation of the proteins contributing to collagen biosynthesis and an overall imbalance in the required levels of proteases (KLK4 and MMP-20) and anti-proteases (anti-thrombin-III, which inhibits KLK-4), essential for optimal mineralization, were observed. Increased albumin levels, probably, contribute to the inhibition of hydroxyapatite crystal growth, resulting in impaired mineralization [11].

While enamel shows a characteristic subsurface lesion, MIH-affected dentin is more homogeneous [13]. However, the study of Heijs et al. [34] showed that in severe cases of MIH, the dentin under hypomineralized enamel is also affected and presents hypocalcemia with few morphological changes but a high frequency of interglobular dentin. The increase in the organic matrix deteriorates the quality of the junction between the dentin and the restorative material. This was observed in specimens of the teeth affected by severe MIH, which was complicated by deep caries and showed the yellow-brown color of demarcations. In permanent teeth, the shape of dentinal tubules within the crown should resemble the letter S [39]. The dentine of specimens, examined in the present study, showed an irregular pattern of dentinal tubules, and in the parts under enamel hypomineralization, the dentin lost its tubular appearance. However, after deproteinization with a 5.25% solution of NaOCl, the dentin morphology was more regular.

Data from the literature clearly show that adhesion to hypomineralized enamel is significantly lower than in healthy enamel due to its porosity, decreased microhardness, unstructured enamel surfaces, and irregular apatite [13,15], which lead to the disruption of bonded margins or even retention loss of the restorations in teeth with MIH [17]. Microleakage can result in marginal discoloration, secondary caries, postoperative sensitivity, and pulpal pathology [29]. Taking the 10 times higher than in sound teeth susceptibility to caries in hypomineralized molar teeth affected by post-eruptive enamel breakdown [40] and the presence of subclinical pulpitis into consideration [20], failure of the restoration marginal seal may significantly worsen the prognosis of MIH-affected teeth. As altered enamel has increased protein content and presents an irregular etching pattern, the removal of surface protein using 5% sodium hypochlorite was suggested to improve bonding to adhesive materials [17,18,37].

Since sodium hypochlorite (NaOCl) has an ability to remove the organic phase of the smear layer from the dentine, the use of 5.25% NaOCl as a deproteinizing agent prior to acid etching may allow for removing organic elements of both the enamel structure and the acquired pellicle [41]. When sodium hypochlorite comes in contact with organic material, fatty acids react with sodium hydroxide, creating soap and glycerol (saponification reaction, which reduces surface tension), amino acids that react with sodium hydroxide to create salt and water (neutralization reaction), and also reactions with hypochlorous acid that create chloramines and water, which reacts with calcium oxide The hydroxyl ions from formatted calcium hydroxide cause protein breakdown [42,43].

The data of the effect of deproteinization in healthy enamel are inconclusive. Espinosa et al. [41,43], in 2008 and 2010, showed that removing organic content from the normal enamel surface with 5.25% NaOCl as a deproteinizing agent prior to phosphoric acid etching significantly increased the enamel’s retentive surface and enhanced the type 1 and type 2 etching patterns. Similarly, Panchal et al. [44] and Sharma et al. [45] showed that enamel deproteinization improves the quality of following acid etching by the removal of a tenacious acquired pellicle layer, which changes the topographical features significantly from type 3 to type 1 and type 2 etching patterns. These enhanced the shear bond strength of orthodontic brackets to both healthy enamel and those affected with fluorosis [44,45]. By contrast, Ahuja et al. [42] concluded that 5.25% NaOCl enamel deproteinization prior to etching did not grossly alter the surface topographic features of enamel and that etching alone still remains the best method for the pretreatment of enamel.

A meta-analysis of in vitro studies suggested that the bond strength of composite materials was improved by the application of NaOCl prior to enamel etching with phosphoric acid, but it did not improve the SBS of self-etch adhesives to enamel. Additionally, NaOCl had no significant effect when applied to sound enamel after etching [46]. By contrast, Gandhi et al. [19] and Kramer et al. [13] observed no effect of deproteinization before etching on adhesion to MIH-affected enamel, whereas Chay et al. [18] noted that deproteinization after etching was beneficial regardless of MIH severity, and Ekambaram et al. [37] showed that it enhanced bond strength, but only to creamy-white samples. A systematic review published in 2024 demonstrated that there is a moderate level of evidence that the deproteinization of MIH-affected enamel with NaOCl after etching marginally enhances the survival rate and the SBS of direct restorations. Outcomes for these parameters were not provided for dentin [47].

Delagdo et al. [48], in their systematic review, concluded that the data on the influence of the dentin pretreatment with NaOCl was contradictory. Some study results showed the negative effect of deproteinization or lack of advantage in using NaOCl treatment, while other results indicated an increased shear bond strength to dentin depending on the adhesive system composition. However, in the reviewed in vitro studies, NaOCl solutions of various concentrations were used (ranging from 0.5% to 10%), and their application times varied (from 1 min to 1 h) [48]. Nevertheless, deproteinization appears to enhance composite adhesion in the case of teeth with developmental defects, which are characterized by decreased mineral and increased protein contents, such as hypocalcified amelogenesis imperfecta, fluorosis, or MIH [18,37,45,49].

It was also suggested that residual NaOCl and its oxygen by-product may interrupt the polymerization of resin-based adhesives, which reduces bond strength [50]. However, the different mechanisms of bonding to the dentin of the resin composites (based on hybrid layer formation that can be impaired by NaOCl) and of glass ionomers (chemical bonding) should be also considered [51]. The glass ionomer material bonds to the tooth tissue in two stages. In the mechanical interlocking stage, penetration of the glass ionomer cement into the uneven dentin surface takes place due to the GIC polyacid component. The second stage (chemical bonding) takes place on the hydroxyapatite surface. Ionic bonds are formed between calcium ions and carboxylate functional groups of the polyalkenoic acid. This type of bonding was observed in both enamel and dentin [24]. Hardening of the glass ionomer material takes place through acid–base reactions. Acid and glass react with the release of Ca^2^+ and Al^3^+ ions, which then combine with polyalkenoic acid chains. If the glass ionomer is modified with resin, additional 2-hydroxyethyl methacrylate (HEMA) polymerization reactions are observed. This modification requires activation with light. However, the biocompatibility of the material is compromised by the presence of the resin component, which is cytotoxic to the cells of the pulp [24]. Additionally, the use of a polymerization lamp can increase the temperature in the pulp area by up to 4.9 °C [52]. This also is one of the reasons for replacing the light-cured resin coating agent (Equia Forte Coat) with petroleum jelly in the case of restoring severely MIH-affected molar teeth with deep caries in children. In addition, it has been shown that the use of Equia Forte Coat reduced the “blast effect” and caused the release of ions from the filling into the oral environment at lower levels for 15 days [53,54,55].

GIGs can adhere to tooth substrates covered in a smear layer without any pretreatment because they contain poly(acrylic acid). However, the smear debris remaining in the adhesive interface becomes an obstacle to ideal bond formation [51]. Although, oxidizing solutions can effectively dissolve the organic phase of the dentin smear layer, there are limited literature sources on the comprehensive in vitro analysis of the influence of deproteinization on glass ionomer bonding. We have not found any other study testing the effect of deproteinization on MIH-affected teeth prior to restoring with a glass hybrid. The study of Justus et al. [56] demonstrated that enamel deproteinizing before 37% phosphoric acid etching and wetting of the enamel surface increased the mean bracket shear bond strength of an RMGIC resin-modified glass-ionomer cement(Fuji Ortho LC) by almost 70% (from 5.7 to 9.6 MPa).

An in vitro study of Hajizadeh et al. [50] assessed the influence of the smear-layer deproteinization using 5% NaOCl solution, followed by etching with phosphoric acid, on resin-modified glass-ionomer bonding to dentin. The authors concluded that NaOCl deproteinization probably led to a mineral surface rich in hydroxyl carbonate and phosphate groups, which become available for bonding. These had a positive influence of the dentin SBS value of Fuji II LC compared with the ‘no pretreatment’ group, but the application of 10% poly(acrylic acid) gave the best result. It can be probably be explained by the fact that the etching effect of phosphoric acid is considered too strong for the bonding of GIC to dentin, and the excessive demineralization would presumably induce a poorer chemical interaction of GIC within the demineralized dentin substrate [51]. RMGICs, similarly to resin composite, may suffer from the interference of residual oxidizing products on NaOCl-treated dentin surfaces with their polymerization, which negatively affect bond strength [57]. This was also confirmed by the study of Paing et al. [51], where 40 ppm solution of hypochlorous acid (HOCl) was used for deproteinization. However, the study proved that smear-layer deproteinization with HOCl can enhance the dentin bonding of conventional GIC (Fuji IX). According to the authors, this might be attributed to the enhancement of the chemical interaction of GIC with hydroxyapatite due to an increased mineral–organic ratio on the HOCl-treated dentin surface [51].

The results of the current study are preliminary findings to signalize that the deproteinization of hypomineralized tissue may influence the marginal gap of Equia Forte glass ionomer. Further laboratory studies are needed to analyze the effect of NaOCl or alternative deproteinizing agents such as HOCl or Papacarie on the surface area of MIH-affected enamel and dentin in the context of marginal adaptation and the adhesion of glass hybrid materials.

### Study Limitation

The limitation of the present study was a small sample size per group. No power test was performed. So, the study can be not adequately powered to detect small-to-moderate group differences for marginal crevices. Additionally, the enamel and dentin were of different amounts in the specimens. These can be justified by the difficulty in obtaining good quality specimens from the extracted teeth with hypomineralization. MIH-affected first permanent molars, extracted due to poor prognosis, usually have extensive post-eruptive breakdown and atypical restorations, leaving a small amount of remaining hard dental tissue. In addition, due to a weakened structure, their crowns very often get fractured during extraction.

## 5. Conclusions

The results of the specimens assessment using a stereoscopic microscope showed that the deproteinization with 5.25% NaOCl solution can favorably affect the marginal adaptation of glass hybrid filling material (Equia Forte HT) to hard dental tissues affected by hypomineralization. However, SEM assessment rejected the hypothesis that pretreatment with 5.25% NaOCl reduces the marginal crevices between the GIC and MIH-affected hard tissues, since cavity surface deproteinization only resulted in a statistically insignificant lower median value of the marginal gap. Further studies are needed to confirm a favorable effect of deproteinization on the marginal adaptation of a glass hybrid.

## Figures and Tables

**Table 1:** Measurements of marginal crevice between Equia Forte HT glass ionomer material and the tooth surface observed in a stereoscopic microscope.

	Standard-Prepared Samples	Deproteinized Samples
Mean	17.33 [μm]	8.81 [μm]
Median	12.61 * [μm]	6.78 * [μm]
Minimum	7.37 [μm]	2.58 [μm]
Maximum	50.19 [μm]	17.46 [μm]
Standard deviation	13.46	4.553
Coefficient of variation	0.776	0.516
Skewness	1.524	0.545
Kurtosis	0.838	−0.893
Range Q1–Q3	10.21	7.03

* Statistically significant differences (Z = 2.652, *p* = 0.008).

**Table 2:** Measurements of marginal crevice measurements between hybrid glass material (Equia Forte HT) and the tooth surface observed in SEM.

	Standard-Prepared Samples	Deproteinized Samples
Mean	237.22 [μm]	97.19 [μm]
Median	156.75 [μm]	69.41 [μm]
Minimum	41.74 [μm]	41.07 [μm]
Maximum	591.82 [μm]	194.65 [μm]
Standard deviation	195.24	76.331
Coefficient of variation	0.825	0.785
Skewness	0.541	1.552
Kurtosis	−1.133	0.959
Range Q1–Q3	348.76	70.47

## Data Availability

The original contributions presented in this study are included in the article. Further inquiries can be directed to the corresponding authors.

## Appendix A

**Table A1:** Measurements of marginal crevice between hybrid glass ionomer material (Equia Forte HT) and the tooth surface [μm] observed in a stereoscopic microscope.

Standard-Prepared Samples (n = 17)	Deproteinized Samples (n = 17)
7.37	2.58
8.57	5.5
8.4	3.19
13.52	16.39
38.17	13.21
50.19	11.27
43.84	12.8
15.73	7.84
10.95	6.45
8.79	4.21
9.34	6.54
7.45	6.66
13.81	6.78
12.61	6.66
22.05	17.46
15.04	8.01
8.91	14.28

**Table A2:** Measurements of marginal crevice between hybrid glass ionomer material (Equia Forte HT) and the tooth surface [μm] observed in SEM.

Standard-Prepared Samples (n = 10)	Deproteinized Samples (n = 10)
183.79	54.42
112.01	64.91
41.74	73.9
55.24	194.65
48.82	273.78
129.74	41.07
398.69	73.9
397.3	97.02
413.51	45.43
591.35	52.8

## References

[1] Lygidakis N.A., Garot E., Somani C., Taylor G.D., Rouas P., Wong F.S.L. (2022). Best clinical practice guidance for clinicians dealing with children presenting with molar-incisor-hypomineralisation (MIH): An updated European Academy of Paediatric Dentistry policy document. Eur. Arch. Paediatr. Dent..

[2] Lopes L.B., Machado V., Mascarenhas P., Mendes J.J., Botelho J. (2021). The prevalence of molar-incisor hypomineralization: A systematic review and meta-analysis. Sci. Rep..

[3] Lygidakis N.A., Wong F., Jälevik B., Vierrou A.M., Alaluusua S., Espelid I. (2010). Best clinical practice guidance for clinicians dealing with children presenting with molar-incisor-hypomineralisation (MIH): An EAPD policy document. Eur. Arch. Paediatr. Dent..

[4] Weerheijm K.L., Jalevik B., Alaluusua S. (2001). Molar-incisor hypomineralisation. Caries Res..

[5] Ammar N., Fresen K.F., Schwendicke F., Kühnisch J. (2025). Epidemiological trends in enamel hypomineralisation and molar-incisor hypomineralisation: A systematic review and meta-analysis. Clin. Oral Investig..

[6] Sluka B., Held U., Wegehaupt F., Neuhaus K.W., Attin T., Sahrmann P. (2024). Is there a rise of prevalence for molar incisor hypomineralization? A meta-analysis of published data. BMC Oral Health.

[7] Sezer B., Çarıkçıoğlu B. (2024). Treatment strategies for incisors of children affected by molar incisor hypomineralization: A narrative review. Oral.

[8] Elhennawy K., Manton D.J., Crombie F., Zaslansky P., Radlanski R.J., Jost-Brinkmann P.G., Schwendicke F. (2017). Structural, mechanical and chemical evaluation of molar-incisor hypomineralization-affected enamel: A systematic review. Arch. Oral Biol..

[9] Marouane O., Manton D.J. (2022). The use of transillumination in mapping demarcated enamel opacities in anterior teeth: A cross-sectional study. Int. J. Paediatr. Dent..

[10] Fagrell T.G., Dietz W., Jälevik B., Norén J.G. (2010). Chemical, mechanical and morphological properties of hypomineralized enamel of permanent first molars. Acta Odontol. Scand..

[11] Mukhtar U., Goyal A., Luthra-Guptasarma M., Gauba K., Kapur A., Thakur A.K. (2022). Label-free quantitative proteomics reveals molecular correlates of altered biomechanical properties in molar incisor hypomineralization (MIH): An in vitro study. Eur. Arch. Paediatr. Dent..

[12] Bozal C.B., Kaplan A., Ortolani A., Cortese S.G., Biondi A.M. (2015). Ultrastructure of the surface of dental enamel with molar incisor hypomineralization (MIH) with and without acid etching. Acta Odontol. Latinoam..

[13] Krämer N., Bui Khac N.N., Lücker S., Stachniss V., Frankenberger R. (2018). Bonding strategies for MIH-affected enamel and dentin. Dent. Mater..

[14] William V., Burrow M.F., Palamara J.E., Messer L.B. (2006). Microshearbond strength of resin composite to teeth affected by molar hypomineralization using 2 adhesive systems. Pediatr. Dent..

[15] Lygidakis N.A., Chaliasou A., Siounas G. (2003). Evaluation of composite restorationsin hypomineralised permanent molars: A four-year clinical trial. Eur. J. Paediatr.Dent..

[16] Fayle S.A. (2003). Molar incisor hypomineralisation: Restorative management. Eur. J.Paediatr. Dent..

[17] Sönmez H., Saat S. (2017). A Clinical Evaluation of Deproteinization and different cavity designs on Resin Restoration Performance in MIH-affected molars: Two-year results. J. Clin. Pediatr. Dent..

[18] Chay P.L., Manton D.J., Palamara J.E. (2014). The effect of resin infiltration and oxidative pre-treatment on microshear bond strength of resin composite to hypomineralised enamel. Int. J. Paediatr. Dent..

[19] Gandhi S., Crawford P., Shellis P. (2012). The use of a ‘bleach-etch-seal’ deproteinization technique on MIH affected enamel. Int. J. Paediatr. Dent..

[20] Rodd H.D., Graham A., Tajmehr N., Timms L., Hasmun N. (2021). Molar incisor hypomineralisation: Current knowledge and practice. Int. Dent. J..

[21] Jälevik B., Klingberg G.A. (2002). Dental treatment, dental fear and behaviour management problems in children with severe enamel hypomineralization of their permanent first molars. Int. J. Paediatr. Dent..

[22] Weerheijm K.L. (2003). Molar incisor hypomineralization. Eur. J. Paediatr. Dent..

[23] Kielbassa A.M., Oehme E.P., Shakavets N., Wolgin M. (2021). In vitro wear of (resin-coated) high-viscosity glass ionomer cements and glass hybrid restorative systems. J. Dent..

[24] Sidhu S.K., Nicholson J.W. (2016). A review of glass-Ionomer cements for clinical dentistry. J. Funct. Biomater..

[25] Pinto N.S., Jorge G.R., Vasconcelos J., Probst L.F., De-Carli A.D., Freire A. (2023). Clinical efficacy of bioactive restorative materials in controlling secondary caries: A systematic review and network meta-analysis. BMC Oral Health.

[26] Inchingolo A.M., Inchingolo A.D., Viapiano F., Ciocia A.M., Ferrara I., Netti A., Dipalma G., Palermo A., Inchingolo F. (2023). Treatment Approaches to Molar Incisor Hypomineralization: A Systematic Review. J. Clin. Med..

[27] Ong J., Yap A.U., Hong J.Y., Eweis A.H., Yahya N.A. (2018). Viscoelastic properties of contemporary bulk-fill restoratives: A dynamic-mechanical analysis. Oper. Dent..

[28] EQUIA Forte HT Comprehensive Guide_New1.Indd—Gc.Dental. https://www.gc.dental/america/products/operatory/glass-hybrid-restoratives/equia-forte-ht.

[29] Singh S., Goel D., Awasthi N., Khandelwal D., Sharma A., Patil S. (2021). Comparative evaluation of marginal Integrity of three esthetic restorative materials—An in-vitro study. Contemp. Clin. Dent..

[30] Miletić I., Baraba A., Krmek S.J., Perić T., Marković D., Basso M., Ozkaya C.A., Kemaloglu H., Turkun L.S. (2024). Clinical performance of a glass-hybrid system in comparison with a resin composite in two-surface class II restorations: A 5-year randomised multi-centre study. Clin. Oral Investig..

[31] Grossi J.A., Cabral R.N., Ribeiro A.P.D., Leal S.C. (2018). Glass hybrid restorations as an alternative for restoring hypomineralized molars in the ART model. BMC Oral Health.

[32] Durmus B., Sezer B., Tugcu N., Caliskan C., Bekiroglu N., Kargul B. (2021). Two-year survival of high-viscosity glass ionomer in children with molar incisor hypomineralization. Med. Princ. Pract..

[33] Sezer B., Şen Yavuz B., İşseven C.İ., Tuğcu N., Çalışkan C., Durmuş B., Kargül B. (2025). Six-year survival and clinical performance of glass hybrid restorations following selective caries removal in teeth with molar incisor hypomineralization: A prospective cohort study. Clin. Oral Investig..

[34] Heijs S.C., Dietz W., Norén J.G., Blanksma N.G., Jälevik B. (2007). Morphology and chemical composition of dentin in permanent first molars with the diagnose MIH. Swed. Dent. J..

[35] Denis M., Atlan A., Vennat E., Tirlet G., Attal J.P. (2013). White defects on enamel: Diagnosis and anatomopathology: Two essential factors for proper treatment (part 1). Int. Orthod..

[36] Mubarak R., Ali W. (2010). Morpho-histological and biochemical study of enamel in sound and hypomineralized permanent first molars. Egypt. Dent. J..

[37] Ekambaram M., Anthonappa R.P., Govindool S.R., Yiu C.K.Y. (2017). Comparison of deproteinization agents on bonding to developmentally hypomineralized enamel. J. Dent..

[38] Wang C., Ou Y., Zhang L., Zhou Z., Li M., Xu J., Fan J., Fu B., Hannig M. (2018). Effects of regional enamel and prism orientations on bovine enamel bond strength and cohesive strength. Eur. J. Oral Sci..

[39] Wang R., Niu L., Li Q., Liu Q., Zuo H. (2017). The peritubular reinforcement effect of porous dentine microstructure. PLoS ONE.

[40] Pitiphat W., Luangchaichaweng S., Pungchanchaikul P., Angwaravong O., Chansamak N. (2014). Factors associated with molar incisor hypomineralization in Thai children. Eur. J. Oral Sci..

[41] Espinosa R., Valencia R., Uribe M., Ceja I., Cruz J., Saadia M. (2008). Enamel deproteinization and its effect on acid etching: An in vitro study. J. Clin. Pediatr. Dent..

[42] Ahuja B., Yeluri R., Baliga S., Munshi A.K. (2010). Enamel deproteinization before acid etching-a scanning electron microscopic observation. J. Clin. Pediatr. Dent..

[43] Espinosa R., Valencia R., Uribe M., Ceja I., Cruz J., Saadia M. (2010). Resin Replica in enamel deproteinization and its effect on acid etching. J. Clin. Pediatr. Dent..

[44] Panchal S., Ansari A., Jain A.K., Garg Y. (2019). Effects of different deproteinizing agents on topographic features of enamel and shear bond strength—An in vitro study. J. Orthod. Sci..

[45] Sharma R., Kumar D., Verma M. (2017). Deproteinization of fluorosed enamel with sodium hypochlorite enhances the shear bond strength of orthodontic brackets: An in vitro study. Contemp. Clin. Dent..

[46] Fernández-Barrera M.Á., da Silva A.F., Pontigo-Loyola A.P., Zamarripa-Calderón J.E., Piva E., Cuevas-Suárez C.E. (2021). The effect of deproteinizing agents on bond strength of resin-based materials to enamel: A systematic review and meta-analysis of in vitro studies. J. Adhes. Dent..

[47] Voinot J., Bedez M. (2024). Pretreatments to bonding on enamel and dentin disorders: A systematic review. Evid. Based Dent..

[48] Delgado A.H.S., Belmar Da Costa M., Polido M.C., Mano Azul A., Sauro S. (2022). Collagen-depletion strategies in dentin as alternatives to the hybrid layer concept and their effect on bond strength: A systematic review. Sci. Rep..

[49] Saroğlu I., Aras S., Oztaş D. (2006). Effect of deproteinization on composite bond strength in hypocalcified amelogenesis imperfecta. Oral Dis..

[50] Hajizadeh H., Ghavamnasiri M., Namazikhah M.S., Majidinia S., Bagheri M. (2009). Effect of different conditioning protocols on the adhesion of a glass ionomer cement to dentin. J. Contemp. Dent. Pract..

[51] Paing S.Y., Tichy A., Hosaka K., Nagano D., Nakajima M., Tagami J. (2020). Effect of smear layer deproteinization with HOCl solution on the dentin bonding of conventional and resin-modified glass-ionomer cements. Eur. J. Oral Sci..

[52] Gorseta K., Glavina D., Skrinjaric T., Czarnecka B., Nicholson J.W. (2016). The effect of petroleum jelly, light-cured varnish and different storage media on the flexural strength of glass ionomer dental cements. Acta Biomater. Odontol. Scand..

[53] Bueno L.S., Silva R.M., Magalhães A.P.R., Navarro M.F.L., Pascotto R.C., Buzalaf M.A.R., Nicholson J.W., Sidhu S.K., Borges A.F.S. (2019). Positive correlation between fluoride release and acid erosion of restorative glass-ionomer cements. Dent. Mater..

[54] Habib S. (2020). Fluoride releasing/recharging ability of bulk-fill and resin modified glass ionomer Cements after the application of different surface coating agents: An in-vitro study. Adv. Dent. J..

[55] Banic Vidal L.S., Veček N.N., Šalinović I., Miletić I., Klarić E., Jukić Krmek S. (2023). Short-term fluoride release from ion-releasing dental materials. Acta Stomatol. Croat..

[56] Justus R., Cubero T., Ondarza R., Morales F. (2010). New technique with sodium hypochlorite to increase bracket shear bond strength of fluoride-releasing resin-modified glass ionomer Cements: Comparing Shear Bond Strength of Two Adhesive Systems with enamel surface deproteinization before etching. Semin. Orthod..

[57] Sekhar A., Anil A., Thomas M.S., Ginjupalli K. (2017). Effect of various dentin disinfection protocols on the bond strength of resin modified glass ionomer restorative material. J. Clin. Exp. Dent..

